# Notes and News

**Published:** 1899-09-02

**Authors:** 


### Notes and News.

(Collected and Communicated.)

The managers of the *Times* are about to bring out the " Century Dictionary," and propose to devote £1 1s. for each copy sold to the Prince of Wales's Hospital Fund. Subscribers will have their names entered as contributors to the Fund.

At Exeter an outbreak of typhoid fever has occurred. The cause has been traced to the consumption of raw cockles at Exmouth, which were partaken of by a party of excursionists. The cockles at Exmouth are reported to be collected from mud-flats near the mouth of a sewer. Nearly forty persons have been attacked with the disease.

The conditions of the will of the late Baroness de Hirsch, respecting which so many fabulous stories have been afloat, have now been made known. The Baroness has not made any legacies direct to hospitals, and her charities are solely for the benefit of the Jewish communities in several countries.

There seems to have been an extraordinary recrudescence of plague in various parts of India lately. Poona has suffered especially severely, but several other places have been attacked. We may judge how severely Poona has been affected from the statement that if the same death-rate as has obtained in some of the worst weeks were continued throughout the whole year it would mean that 340 out of every 1,000 living would die. Fortunately, such an alarming death-rate does not often last for long.

The reports from various other quarters where plague actually exists, or is suspected, are not very encouraging. Naturally in Europe attention is principally directed to Portugal. At Oporto, around which a military cordon has now been established, over 50 cases have been recorded, and several cases have been already notified in the suburbs. At Madrid preparations for the manufacture of anti-plague serum are being hurried on. It is now thought that the epidemic was imported into Portugal by a Greek ship laden with grain, which had touched Alexandria. At Alexandria fresh patients still continue to be attacked, but there is now no rapid increase. In Russia much alarm is still felt, as the patients suspected of suffering from the disease show pulmonary symptoms, which have also been a feature of the epidemic in Portugal.

The latest report of the Government Analytical Laboratory states that a large number of the medical preparations which were examined for the Army Medical Department were found to be markedly inferior to the standard of the British Pharmacopœia.

The Sanitary Association of Holbeck and Hunslet, in Yorkshire, is proposing to open a small sanatorium for consumptives on the hills above Otley. Mr. D'Arcy Wyvill has offered the association a farmhouse rent free, and Dr. Bennett, the medical officer for health at Otley, has promised his services.

Newcastle is fortunate in having received so much generous support towards the new infirmary scheme. Mr. John Hall's bequest of £100,000 was contingent upon the collection of a similar sum from the public within three years from the time the offer was made. The condition is now fulfilled, as Mr. J. Eno, formerly of Newcastle, has just made a donation of £8,500 for the purpose of completing the £100,000 contributed by the public.

The Duke of Connaught visited Dundee last week for the purpose of opening the Victoria Hospital for Incurables, and to unveil a statue of the Queen. The statue, the Victoria Hospital, and a maternity hospital serve to commemorate the Queen's Jubilee on behalf of the people of Dundee. The Hospital for Incurables is situated on an elevated site in a fine public park, and has cost £50,000.

In consequence of the discovery of the malaria-carrying mosquito by Dr. Donald Ross, and the need of another scientist to be sent out to Sierra Leone, the Liverpool School of Tropical Medicine has selected Dr. Fielding Ould for this purpose. He expects to leave Liverpool for Sierra Leone on Saturday, September 2nd. Dr. Ould has been working for some time with Professor Boyce in the pathological department of University College, Liverpool.

The City Hospital, Aberdeen, has just undergone considerable improvements. A new block has been added northwards near the administration block. It is a two-storeyed elevation, in keeping with the rest of the building. It is devoted to administration purposes with the exception of a small part reserved for the reception and discharge of patients. The additional accommodation has enabled other parts of the building to be improved. The cost has been between £4,000 and £5,000.

It is a curious thing that in some parts of South Wales, not so far from the very districts from which it is proposed to bring water to London, the people are at the present time almost worse off for water than in any part of England. Take the year round there is water enough; it is all a question of storage, and this is deficient. Here we have an instance of districts whose annual rainfall approaches 100 inches per annum, *i.e.*, between three and four times the average rainfall of England, suffering all the discomforts of an almost complete deprivation of their water supply. It is an admirable example of the degree to which a civilised community becomes dependent upon the maintenance of purely artificial conditions, and of the necessity of a provident and far-seeing civic administration.

